# A Rare Case of Fatal Hemorrhagic Stroke in a Young Female with Early Mixed Connective Tissue Disease

**DOI:** 10.1155/2021/5321438

**Published:** 2021-10-28

**Authors:** James R. Agapoff IV

**Affiliations:** Department of Psychiatry, University of Hawai‘i at Mānoa, John A. Burns School of Medicine, Honolulu, HI, USA

## Abstract

Mixed connective tissue disease (MCTD) often presents as a slow progressive illness with low morbidity and mortality. Serious central nervous system disease is uncommon, and fatal outcomes are rarely seen. Here, we report a rare case of fatal hemorrhagic stroke in a 43-year-old female with a rapidly progressive MCTD. She presented to primary care with a history of headaches, visual disturbances, and unprovoked lower extremity swelling and pain. A rheumatological workup showed positive antinuclear (ANA) and ribonucleoprotein (RNP) antibodies. Magnetic resonance imaging (MRI) found a 12 mm hemorrhage along a cortical sulcus of the right frontal lobe, and a follow-up magnetic resonance angiography (MRA) and ophthalmological exam showed no definitive signs of vasculitis. Over the course of her workup, she developed swollen hands, Raynaud's syndrome, myalgias, and synovitis characteristic of evolving MCTD. The patient then began to experience severe headaches over one month. Repeat MRI was ordered, but never completed, and the patient presented to the emergency department (ED) with a severe, right-sided headache, and left-sided visual disturbance. In the ED, she began to display evidence of delirium and seizure activity and became unresponsive. A computerized tomography scan (CT) of the brain showed a right parietal lobe intraparenchymal hemorrhage approximately 5 × 3 × 5 cm in size with secondary mass effect including mid- and hind-brain herniation. Computerized tomography angiography (CTA) of the brain showed signs of large vessel vasculitis. A craniectomy was performed; however, the patient never regained consciousness and died several days later. Vasculitis, while rare in connective tissue diseases, should be aggressively assessed for and managed in patients with any early signs and symptoms of cerebrovascular involvement to prevent fatal outcomes.

## 1. Introduction

Mixed connective tissue disease (MCTD) is an autoimmune condition characterized by overlapping features of several connective tissue diseases including polymyositis, rheumatoid arthritis, systemic lupus erythematosus, and systemic sclerosis in the presence of high levels of RNP antibodies [1]. Of the diagnostic algorithms available, the Alarcon–Segovia's and Kahn's criteria are considered to have highest sensitivity and specificity [2].

MCTD is a rare disease with incidence rates between 0.2 and 2.1 per 100,000 person-years based on several studies [1, 3, 4]. In one nationwide sample, the prevalence of MCTD was 3.8 (95% CI 3.2 to 4.4) per 100,000 adults, the average age was 37.9 years (95% CI: 35.3 to 40.4 years), and the diagnostic ratio of females to males was 3.3 [3].

Compared to other connective tissue diseases, MCTD is generally considered to have lower morbidity and mortality. In one study, mortality to MCTD did not differ from the general population sampled with a mortality ratio of 1.1 (95% CI, 0.4–2.6) [1]. In another study, 15-year survival rates from diagnosis were 88% [5]. Survival rates may be better considering that it often takes a median time of 3.6 years to make the diagnosis from the onset of first symptoms.

Neurological symptoms are uncommon in MCTD; headache, seizure, encephalopathy, aseptic meningitis, and neuropathy are seen in 10–20% of patients. Reports of immune-mediated central nervous system (CNS) vasculitis seen as the initial presentation of MCTD are uncommon [6–8].

Here, we report on the case of a 43-year-old, healthy Caucasian female, with rapidly progressive MCTD who developed neurological symptoms and later died of hemorrhagic stroke associated with probable CNS vasculitis.

## 2. Case Presentation

A 43-year-old healthy Caucasian female presented to primary care with short (7–10 days), recurrent flares of lower extremity tenderness, swelling, and redness over several months. She also reported myalgias in the upper back and neck, and a one-year history of headache with transient, reoccurring curtain-like visual disturbances in her right eye that ophthalmology diagnosed as ocular migraines. A rheumatologic workup was initiated. Labs were positive for an elevated sedimentation rate (ESR) 31 mm/H (normal 0–20 mmH), positive antinuclear antibodies (ANA), and an elevated ribonucleoprotein antibodies (RNP) 2.4 AI (reference <1.0 AI). Double-stranded (DS) DNA 1 IU/mL (<4 = Neg), antichromatin (AC), anti-Smith (SM), SM/RNP, and lyme disease antibodies were negative. Based on these results, the patient received a presumptive diagnosis of undifferentiated connective tissue disease (UCTD), started 20 mg of oral prednisone as needed for flares, and referred to rheumatology.

Two months after her initial evaluation, the patient continued to experience episodes or erythema nodosum of the lower extremities. She also began to experience early signs of Raynaud's phenomenon, chest pain, and visual disturbances concerning for amaurosis fugax. A personal symptom journal kept by the patient indicated transient episodes of fatigue and painful hand/finger swelling with petechiae and purpura. Magnetic resonance imaging (MRI) of the brain was ordered, and repeat labs done by rheumatology showed an elevated ESR, 39 mm/H; RNP, 1.8 AI; CRP, 3.7 (normal <0.8 mg/dL); and a negative antineutrophil cytoplasmic antibody (ANCA), myeloperoxidase antibodies, proteinase-3 antibodies, and ANA. Complement C3, 118 mg/dL (normal 90–180 mg/dL), and C4, 29 mg/dL (normal 16–47 mg/dL), were within normal limits. The patient was referred for further evaluation at an academic medical center.

The MRI showed a 6 mm lacunar infarct within the left cerebellum and a 12 mm hemorrhage along a cortical sulcus of the right frontal lobe. The findings were noted to be “abnormal” for a woman in her early forties, and a further work was recommended for vasculitis and/or connective tissue disease via magnetic resonance angiography (MRA). The MRA and ophthalmological assessment were unremarkable, and cardiology found no evidence of vascular disease on carotid ultrasound and no evidence of exercise-induced myocardial ischemia on stress echocardiogram.

A second rheumatological consult was completed where concern for possible evolving MCTD was raised; however, the diagnosis of UCTD was retained due to the negative ANA. Seven months following her initial presentation, the patient saw neurology where she reported worsening lightheadedness and 6-7/10 bifrontal/apical headaches lasting hours to days over the previous month. Despite the history of arthralgias/myalgias, purpura, chest pain, evidence of cerebral hemorrhage, and initial labs showing nonspecific inflammation and markers of autoimmunity, vasculitis was considered low on the differential due to a lack of a clear prodromal history and changes in cognition. Similarly, neuro-ophthalmology found no definite retinal vasculitis and believed her homonymous right visual field defect to be associated with the brain lesion previously appreciated on MRI and not amaurosis fugax. A nonemergent repeat MRI with contrast and an MR venogram of the brain were ordered by neurology to rule out thrombosis and evaluate for new lesions.

**Figure 1:**
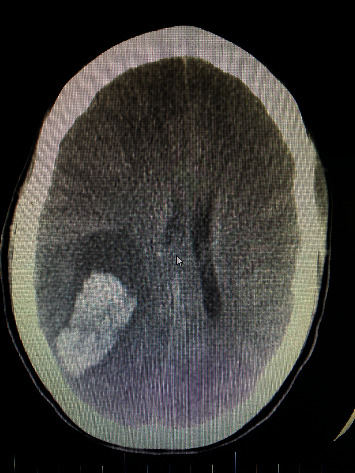
Computerized tomography (CT) axial head without contrast showing intraparenchymal hemorrhage and secondary mass effect.

**Figure 2:**
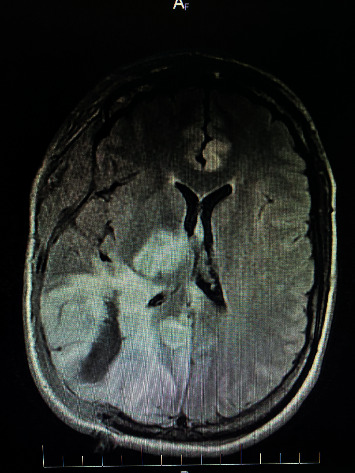
Magnetic resonance imaging (MRI) FLAIR axial head without contrast showing intraparenchymal hemorrhage status after craniectomy.

Approximately two months after her neurology appointment, the patient presented to the emergency room with a severe, right-sided headache with a left-sided visual disturbance. Shortly after arrival, the patient became delirious and exhibited seizure-like activity. A computerized tomography scan (CT) of the brain was conducted that showed a right parietal lobe intraparenchymal hemorrhage approximately 5 × 3 × 5 cm in size with secondary mass effect including mid- and hind-brain herniation (see Figure 1). A CTA showed multifocal narrowing and an abnormal “beaded” contour of the entire cerebral arterial vasculature consistent for vasculitis. Neurosurgical assets were not available, and the patient was transferred to a sister center where craniectomy was performed to evacuate intracerebral hemorrhage and treat intracranial hypertension (see Figure 2). Postoperative prognosis was poor, and palliative measures were initiated. The patient passed away peacefully surrounded by family several days later.

## 3. Discussion

During the patient's clinical course, the diagnosis of MCTD remained in the differential; however, there was early serological and clinical evidence for this condition. The patient was initially ANA and RNP positive and would experience all of clinical criteria for MCTD, save sclerodactyly, over her clinical course including swollen hands, Raynaud's phenomenon, myalgias, and synovitis [1]. Even though the patient's ANA would later be negative, a positive RNP is often considered diagnostic for MCTD when found in isolation of other biomarkers as seen in this patient [9].

Only 10–20% of patients with MCTD experience neurological manifestations, with few case reports known where neurological symptoms were the presenting symptom [6-8]. [6, 7] Similar to the subject of this report, the patients in these studies presented with severe headaches as a prodrome to an immune-mediated CNS vasculitis. Given the progressive nature of MCTD and the short duration (less than one-year) between the onset of this patient's neurological symptoms and her rheumatologic workup, it is likely that this patient's headaches and visual disturbances were an initial manifestation of MCTD and not an isolated case of ocular migraines.

Vasculitis is rare in connective tissue disease, occurring in less than 10% of patients [10] with central nervous system involvement occurring in a much smaller subgroup [11]. In this patient, early signs of small vessel vasculitis included petechiae and purpura on the hands and lower extremities. Evidence of systemic involvement included persistently elevated inflammatory biomarkers (CRP and ESR), amaurosis fugax, and cerebrovascular hemorrhaging noted on MRI. Despite these findings, vasculitis remained low on the differential due to lack of a clear prodrome and frank cognitive deficits.

MCTD often presents as a sequential or staggered overlap of SLE, scleroderma, myositis, and rheumatoid arthritis [12]. This nonlinear presentation requires heightened curiosity of potential evolving pathologies where a clear prodrome is not apparent. Throughout her clinical course, the patient reported mental fogginess that might have been interpreted as “cognitive deficits” in a person with lower baseline intelligence and functional status. And, while the initial MRA of the brain did not show any signs of large vessel disease, it did not capture the distal mid-to-small sized vessels of the brain. This incomplete study could not rule out small dissections or other changes that may have informed clinicians about evolving pathologies including systemic vasculitis.

Had the patient's first MRA shown more definitive signs of vasculitis, her report of new and worsening headaches may have prompted her treatment team to pursue a more aggressive assessment of her symptoms. This case illustrates the importance of considering CNS vasculitis in patients with unexplained, progressive neurologic symptoms and adds support to the case reports by Awad, Matsui, and Graf showing MCTD as a possible cause of severe neurological disease [6–8]. Together, these cases illustrate the importance of rapid and assertive assessment and management of neurological symptoms in persons with suspected connective tissue diseases. Awareness of this link may aid in early diagnosis and initiation of appropriate treatments to improve positive outcomes.

## References

[1] Ungprasert P., Crowson C. S., Chowdhary V. R., Ernste F. C., Moder K. G., Matteson E. L. (2016). Epidemiology of mixed connective tissue disease, 1985–2014: a population-based study. *Arthritis Care & Research*.

[2] Alarcon-Segovia D., Cardiel M. H. (1989). Comparison between 3 diagnostic criteria for mixed connective tissue disease. Study of 593 patients. *Journal of Rheumatology*.

[3] Gunnarsson R., Molberg Ø., Gilboe I.-M., Gran J. T. (2011). The prevalence and incidence of mixed connective tissue disease: a national multicentre survey of Norwegian patients. *Annals of the Rheumatic Diseases*.

[4] Kaipiainen-Seppänen O., Aho K. (1996). Incidence of rare systemic rheumatic and connective tissue diseases in Finland. *Journal of Internal Medicine*.

[5] Hajas A., Szodoray P., Nakken B., Nagy G., Szekanecz Z., Bodolay E. (2013). SAT0190 long-term follow-up of 280 patients with mixed connective tissue disease. *Annals of the Rheumatic Diseases*.

[6] Awad A. M., Stevenson M. (2011). Isolated central nervous system vasculitis associated with antiribonuclear protein antibody. *Case Reports in Neurological Medicine*.

[7] Matsui H., Udaka F., Oda M., Kubori T., Nishinaka K., Kameyama M. (2006). Encephalopathy and severe neuropathy due to probable systemic vasculitis as an initial manifestation of mixed connective tissue disease. *Neurology India*.

[8] Graf W. D., Milstein J. M., Sherry D. D. (1993). Stroke and mixed connective tissue disease. *Journal of Child Neurology*.

[9] Mayo Clinic Laboratories (2021). *Neurology Catalog: RNP Antibodies, IgG, Serum*.

[10] Cozzani E., Gasparini G., Papini M., Burlando M, Drago F, Parodi A (2015). Vasculitis associated with connective tissue diseases. *Giornale italiano di dermatologia e venereologia: organo ufficiale Societa italiana di dermatologia e sifilografia*.

[11] Sharma A., Dhooria A., Aggarwal A., Rathi M., Chandran V. (2016). Connective tissue disorder-associated vasculitis. *Current Rheumatology Reports*.

[12] Pepmueller P. H. (2016). Undifferentiated connective tissue disease, mixed connective tissue disease, and overlap syndromes in rheumatology. *Missouri Medicine*.

