# Erratum to “A Rare Case of Fatal Hemorrhagic Stroke in a Young Female with Early Mixed Connective Tissue Disease”

**DOI:** 10.1155/2022/9762151

**Published:** 2022-02-23

**Authors:** James R. Agapoff IV

**Affiliations:** Department of Psychiatry, University of Hawai‘i, John A. Burns School of Medicine, Honolulu, HI, USA

In the article titled “A Rare Case of Fatal Hemorrhagic Stroke in a Young Female with Early Mixed Connective Tissue Disease” [1], the second paragraph of the Discussion section should be corrected as follows due to an error introduced during the production process.

“Only 10–20% of patients with MCTD experience neurological manifestations, with few case reports known where neurological symptoms were the presenting symptom [6–8]. [6, 7]” to “Only 10–20% of patients with MCTD experience neurological manifestations, with few case reports known where neurological symptoms were the presenting symptom [6–8].”

In addition, Figure 1 is revised as follows, to remove the cursor from the image and include a scale.

## Figures and Tables

**Figure 1:**
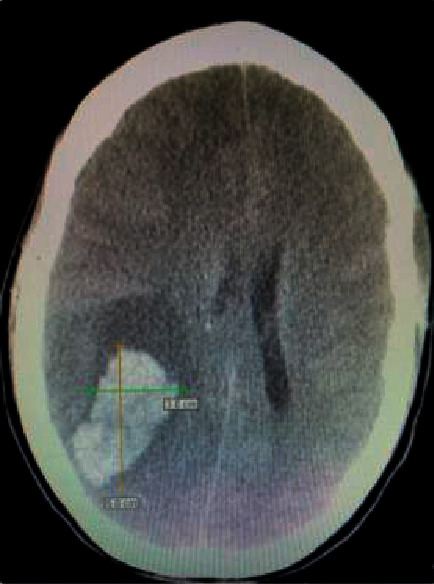
Computerized tomography (CT) axial head without contrast showing intraparenchymal hemorrhage and secondary mass effect.
